# *Bartonella* Species Isolated from Rodents, Greece

**DOI:** 10.3201/eid1005.030430

**Published:** 2004-05

**Authors:** Afrodite Tea, Stella Alexiou-Daniel, Androniki Papoutsi, Anna Papa, Antonis Antoniadis

**Affiliations:** * Aristotelian University of Thessaloniki, Thessaloniki, Greece; † American Hellenic Educational Progressive Association University Hospital, Thessaloniki, Greece

**Keywords:** *Bartonella*, rodents, Greece

**To the Editor**: Domestic cats and human body lice have been identified as the vectors of *Bartonella henselae* and *B. quintana,* respectively, the primary sources of *Bartonella*-associated human diseases (*1*). *Bartonella* species are zoonotic agents that have been isolated from a wide range of mammals in the United States (*2*) and Europe (*3*) and have been associated with human diseases (*4*,*5*).

This study investigated the potential for infection from *Bartonella* species in rodents in northern Greece. The small mammals tested were collected with live traps (*6*). Two sites were surveyed; the first was Nevrokopi, a small town in the Rhodope Mountains near the Greek-Bulgarian border, and the second site included Pramanta, a small village in the Pindos Mountains, and Matsuki, a small village in northwestern Greece. At Nevrokopi, 57 small mammals were captured during 887 trap nights for a success rate of 6.4%. At Pramanta and Matsuki, 13 small mammals were captured during 400 trap nights for a success rate of 3.3%. The 70 captured mammals comprised seven species of rodents. *Apodemus flavicollis* was the most commonly captured species (87%). Blood samples from each of the trapped mammals were frozen in liquid nitrogen in the field and subsequently stored at –70ºC before bacteria isolation. Bacteria isolation was performed as previously described (*7*). One hundred microliters of whole mammalian blood was cultured on heart infusion agar containing 5% rabbit blood (Becton Dickinson, Franklin Lakes, NJ) and incubated in 5% CO_2_ at 35°C for a minimum of 4 weeks. DNA of the putative *Bartonella* cultures was extracted by using QIAamp Tissue Kit (Qiagen GmbH, Hilden, Germany). Polymerase chain reaction (PCR) was performed by using two oligonucleotides specific for the citrate synthase (*glt*A) gene of *B. henselae* Houston 1, primers BhCS 781.p and BhCS 1137.n. Negative and positive controls (double-distilled H_2_O and DNA from cultures of *B. henselae*) were used in each PCR run. Products of the correct size were purified (QIAquick PCR Purification kit, Qiagen GmbH) and sequenced with the same primers, BhCS 781.p and BhCS1137.n., in both directions, with the Cy5/Cy5.5 Dye Primer Cycle Sequencing kit on a Long-Read Tower sequencer (Visible Genetics Inc., Toronto, Canada). Three hundred thirty-eight base-pair sequences of the *glt*A gene were obtained and compared with sequences of other known *Bartonella* species in GenBank by using the nucleotide BLAST program (National Center for Biotechnology Information; Available from: www.ncbi.nlm.nih.gov/BLAST/). Isolates identified as *Bartonella* species were obtained from 21 of the 70 blood cultures. All were isolated from *A. flavicollis,* and one was isolated from *Dryomys nitedula*. In addition, all were isolated from the first site (Nevrokopi village), and one was isolated from the second site (Pramanta village).

Within these 21 *Bartonella* isolates, eight genotypes were found. Among these isolates, one (AY 435102 isolated from *A. flavicollis* trapped in Pramanta), was identical to ma106up strain, isolated from *Microtus agresti* (AF391789); another (AY 435103 isolated from *A. flavicollis* trapped in Nevrokopi), was identical to af82up strain (AF391788), also isolated from *A. flavicollis* (*3*). Both strains ma106up (AF391789) and af82up (AF391788) were isolated in central Sweden (*3*) The rest of *Bartonella* isolates were from mammals trapped in Nevrokopi village and were divided into three phylogenetic groups. The first group, containing 10 isolates (AY435104-AY 435113, isolated from *A. flavicollis*) and representing four novel genotypes, was 98% similar to *B. taylorii* (AF 191502, isolated from *A. sylvaticus*) gltA gene. The second group, consisting of seven isolates that shared the same genotype (AY 435114-AY435120 isolated from *A. flavicollis*), was 99% similar to *B. birtlesii* (AF 204272 isolated from *Apodemus spp.*). The third group consisted of two isolates that shared the same genotype (AY 435121 isolated from *D. nitedula*, and AY435122 isolated from *A.* *flavicollis*); this group was 97% similar to *B. grahamii* strain V2 (Z70016 isolated from *Neomys fodiens*).

This is the first study to identify *Bartonella* in small mammals in Greece. We found that 31.3% of the examined mammals were infected with *Bartonella* spp. The prevalence of culture-positive infections differed between the two sites (20/57 versus 1/13), although both are mountain areas with similar environmental and climatic conditions. A high prevalence of *Bartonella* infection in small mammals also has been described in other countries such as the United States (*7*) and Sweden (*3*), where 42.2% and 16.5% of the collected rodents were infected with *Bartonella* spp., respectively. As indicated in these studies, numerous *Bartonella* species are found in rodents. *A*. *flavicollis* was the most commonly captured species in Sweden (110/236), as well as in Greece (61/70). Identical *Bartonella* strains were isolated from *A*. *flavicollis* and *Microtus agresti* in both countries. Unlike Sweden, where the most frequent genotype was *B. grahamii,* in this study no isolate was identical to any *Bartonella* species known to cause human diseases*.* However, *B. elizabethae* was first isolated from a patient with endocarditis, and nothing was known concerning the organism’s natural history until it was isolated from a rodent captured in Peru (*4*).

The occurrence and distribution of *Bartonella* in European hosts are largely unknown. Given the existence of *Bartonella* spp. in every mammal group examined to date, the diversity of the genus is probably much greater than has been observed among the strains examined to date. In Greece, serologic evidence of human infection with *B. henselae* and *B. quintana* (*8*), has been found and a case of *B. quintana* endocarditis has been established (unpub. data). The public health relevance of *Bartonella* infections in small mammals in Greece compared with other countries remains to be defined.
